# Supplementary material for: Continuous assessment of daily-living gait using self-supervised learning of wrist-worn accelerometer data
Source: NPJ Digit Med. 2026 Mar 12;9:338. doi: 10.1038/s41746-026-02528-2 (PMC13121596; doi:10.1038/s41746-026-02528-2)
Supplement: Supplementary file 1 — Supplementary material [file 41746_2026_2528_MOESM1_ESM.pdf]

Supplementary material

Table S1. Comparison of ElderNet performance with state-of-the-art and supervised models for gait speed estimation across different test sets.

Model	Test Set	MAE (cm/s)	RMSE (cm/s)	MAPE (%)	R^2	ICC
ElderNet	Dataset 3	8.8 [6.53 11.02]	10.7 [8.59 13.75]	12.73 [9.45 15.58]	0.74 [0.47 0.80]	0.87 [0.78 0.92]
	Dataset 4	8.3 [6.53 11.79]	11.4 [8.85 14.49]	8.22 [6.66 11.04]	0.72 [0.57 0.81]	0.87 [0.82 0.92]
Soltani et al.	Dataset 3	16.3 [12.12 18.17]	19.0 [13.99 22.30]	21.27 [17.19 26.70]	0.20 [-0.8 0.6]	0.62 [0.43 0.78]
	Dataset 4	12.9 [9.73 15.13]	14.8 [12.89 17.67]	11.39 [8.86 15.23]	0.68 [0.42 0.73]	0.84 [0.72 0.87]
Supervised	Dataset 3	14.1 [11.33 18.06]	17.4 [14.92 22.21]	21.63 [16.49 31.11]	0.23 [-0.22 0.58]	0.59 [0.38 0.72]
	Dataset 4	13.8 [9.58 16.51]	17.9 [12.91 19.80]	15.85 [9.38 17.23]	0.53 [0.38 0.65]	0.74 [0.68 0.81]

Performance is reported at the subject level as the median (with the 25th and 75th percentiles in brackets). The internal test set (Dataset 3) was the Mobilise-D TVS set, which included 18 participants (20% of the dataset). The external test set (Dataset 4) included 11 healthy young adults.

Table S2. Device specifications across datasets.

Dataset #	Accelerometer Device	Sampling rate (Hz)*	Range (g)
**Dataset 1**	Axivity AX3	100	±8
**Dataset 2**	Axivity AX3, GENEActiv	Axivity: 50; GENEActiv: 40	±8
**Dataset 3**	INDIP	100	±16
**Dataset 4**	INDIP	100	±16

*All signals were resampled to 30 Hz prior to model input.

Table S3. Hyperparameter search space used for ElderNet fine-tuning in gait quality estimation.

Hyperparameter	Values
Learning rate	1e-5, 5e-5, 1e-4, 5e-4, 1e-3
Batch size	64, 128, 256, 512, 1024
Number of layers (regression head)	0-3
Batch norm (for regression layers)	Yes/No
Weight Decay	0, 0.01, 0.1

Table S4. Summary of gait quality and physical activity features.

Measure	Extraction Level*	Unit	Summary Statistics Computed	Distribution Features**
**Gait Measures**				
Gait Speed	10-sec window, Walking bout	cm/s	Median, Mean, Std, Percentiles (10th–90th), Kurtosis, Skewness, Range	Histogram bins (10 bins across 0–180 cm/s)
Stride Length	10-sec window, Walking bout	cm	As above	Histogram bins (10 bins across 0–200 cm)
Cadence	10-sec window, Walking bout	Steps per minute	As above	Histogram bins (10 bins across 40–160 steps/min)
Stride Regularity (Model-based)	10-sec window, Walking bout,	A.U (0-1)	As above	Histogram bins (10 bins across 0–1);
Stride Regularity (Signal Processing)	10-sec window, Walking bout	A.U (0-1)	As above	Histogram bins (10 bins across 0–1)
**Physical Activity Measures**				
Average magnitude of the acceleration signal	Day-level and Walking bout level	m/s²	Median, Mean, Std, Percentiles, Kurtosis, Skewness, Range	—

Standard deviation of the acceleration magnitude signal	Day-level and Walking bout level	m/s²	As above	—
Minimum of the acceleration magnitude signal	Day-level and Walking bout level	m/s²	As above	—
Maximum of the acceleration magnitude signal	Day-level and Walking bout level	m/s²	As above	—

***Extraction Level**:

1. 10-second window level: Statistics were computed over overlapping windows (10-second windows, with 9-second overlap).
2. Walking bout level: Windows were aggregated over contiguous bouts of walking activity.
3. Day-level: Aggregated features computed over the entire day.

****Distribution Features**:

The global ranges used to compute the histogram-based distribution features were determined by combining empirical observations from our dataset with normative values reported in the literature[1,2]. For each gait measure, the observed values were divided into ten equal-width bins across a predefined range. For example, gait speed was set between 0 and 180 cm/s—reflecting the upper limits of walking speeds typically observed in older adults—while cadence was bounded between 40 and 160 steps per minute. Stride length was assigned a range of 0–200 cm, and both the model-based and signal processing–based stride regularity metrics were normalized between 0 and 1. These histogram features were computed separately at two levels: each bin feature represents the number of 10-second windows (where each window contributes one point) or aggregated bouts (where each bout contributes one point) during which the participant's measure falls within that interval (e.g., the bin covering 60–80 cm/s indicates the count of windows or bouts with gait speeds in that range).

Note: Histogram-bin features represent counts of windows or bouts within specific ranges and may partially reflect gait quantity (e.g., total walking time) in addition to gait quality

characteristics. Histogram counts were not normalized by total walking time unless otherwise specified.

Table S5. Hyperparameter Ranges for the XGBoost Classifier

**Hyperparameter**	**Values**
n_estimator	10, 50, 150, 300, 500
learning_rate	0.001, 0.005, 0.01, 0.05, 0.1, 0.2
subsample	0.1, 0.3, 0.6, 0.9
colsample_bytree	0.5, 0.7, 0.8, 0.9
gamma	0, 0.1, 0.2, 0.3, 0.5
alpha	0, 0.01, 0.1, 1
lambda	1, 2, 3

All hyperparameters follow the sklearn-compatible API of xgboost.XGBClassifier. Hyperparameter optimization was conducted using RandomizedSearchCV from scikit-learn with 100 iterations.

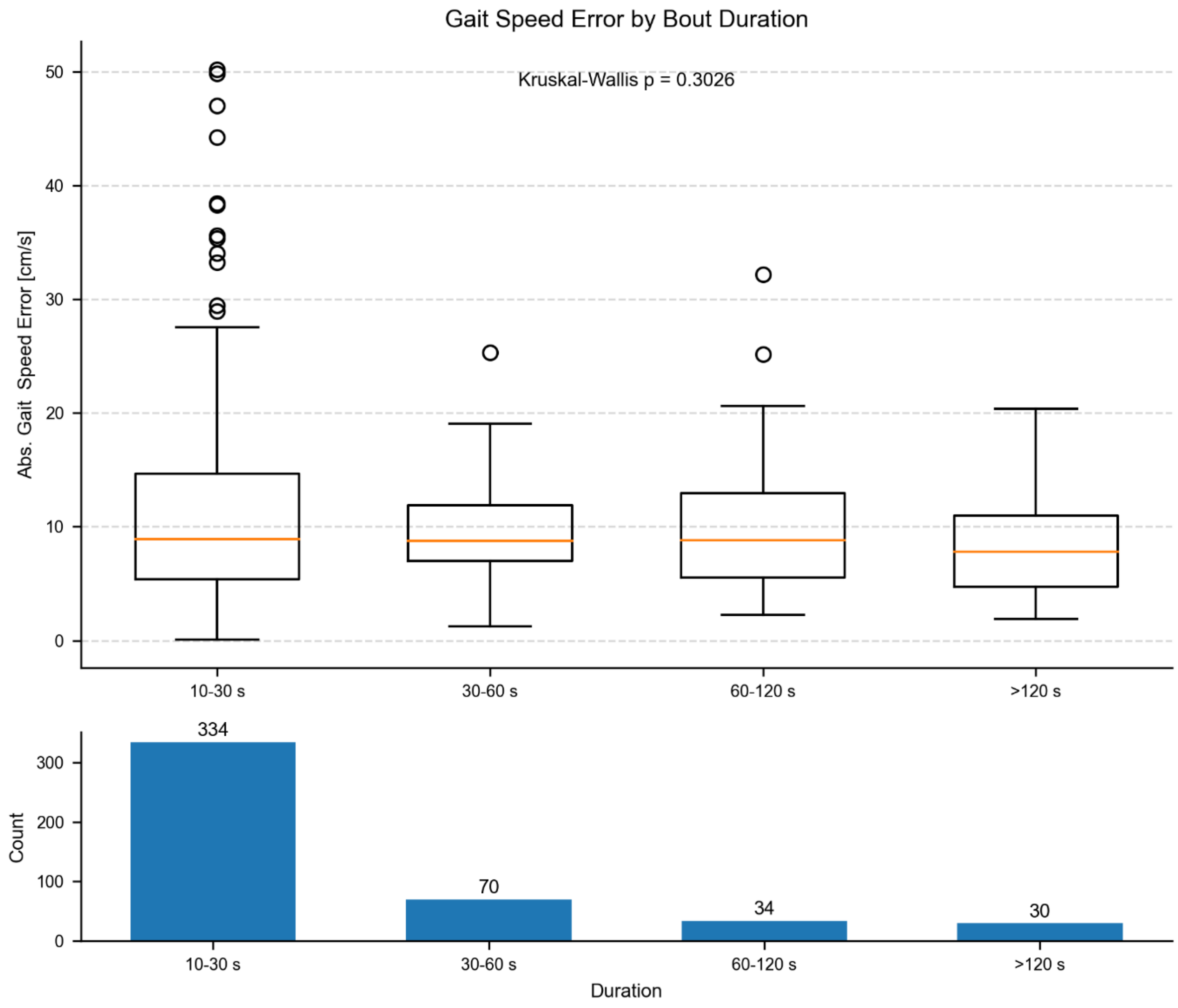

Figure S1. Gait speed error by bout duration. Top: Boxplots of absolute error (cm/s) in Mobilise-D TVS test set (Dataset 3), with outliers (Kruskal-Wallis p = 0.30). Bottom: Bout counts per category.

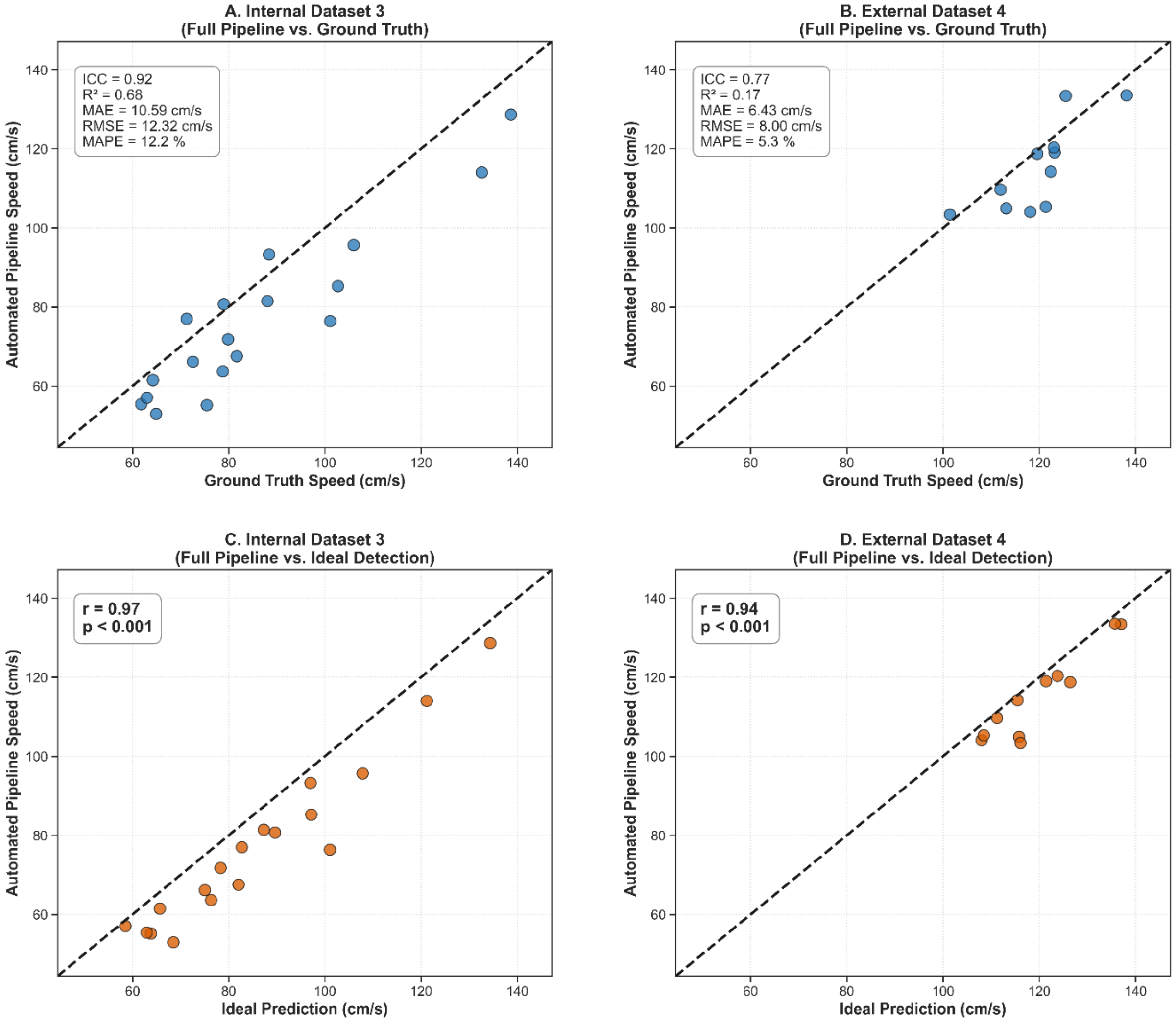

Figure S2. Real-world validity and robustness of the automated gait speed pipeline across internal and external datasets. **(A, B)** Scatter plots illustrating the predictions of the fully automated pipeline (y-axis) compared to ground-truth reference values (x-axis). Panel **(A)** presents the Internal Validation (Dataset 3), while Panel **(B)** presents the External Validation (Dataset 4). The dashed line represents the identity line (y=x). The high Intraclass Correlation Coefficients (ICC) and low error metrics (MAE, RMSE) in both datasets confirm the system's accuracy in fully automated settings. **(C, D)** Scatter plots assessing the specific impact of the automated gait detection step. These panels compare the gait speed calculated by the fully automated pipeline (y-axis) against an "ideal" prediction derived from manually annotated gait bouts (x-axis). Panel **(C)** corresponds to the Internal Dataset, and Panel **(D)** to the External Dataset. The near-perfect Pearson correlation coefficients (r) and statistical significance ($p < 0.001$) demonstrate that the automated detection step introduces negligible distortion to the final gait speed estimates compared to manual segmentation.

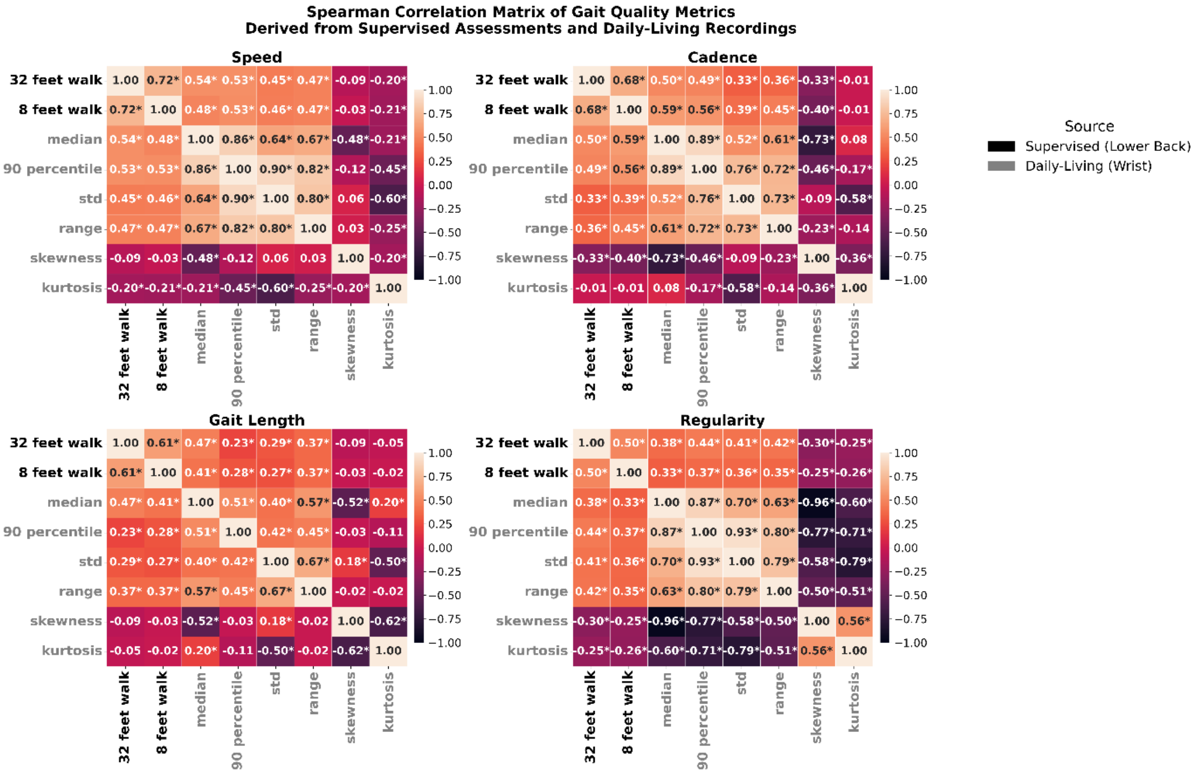

Figure S3. Pairwise correlations between gait measures of speed, cadence, gait length, and regularity obtained from supervised assessments (black labels: 32 feet walk, 8 feet walk) and daily living recording (grey labels: "median," "90 percentile", "std," "range," "skewness"). Each cell shows the Spearman correlation coefficient of the two variables. Diagonal cells are self-correlations (1.00). An asterisk ("*") indicates statistical significance ($p < 0.05$) after Bonferroni correction. While some daily-living metrics correlate with supervised assessments, much of the variance remains unexplained, suggesting that wrist-derived measures reflect supervised performance but also capture additional real-world gait characteristics.

References

1. Hollman, J. H., McDade, E. M. & Petersen, R. C. Normative spatiotemporal gait parameters in older adults. Gait Posture 34, 111–118 (2011).

2. Studenski, S. et al. Gait Speed and Survival in Older Adults. JAMA 305, 50–58 (2011).
